# Impact of Physical Activity and Exercise on the Epigenome in Skeletal Muscle and Effects on Systemic Metabolism

**DOI:** 10.3390/biomedicines10010126

**Published:** 2022-01-07

**Authors:** Julio Plaza-Diaz, David Izquierdo, Álvaro Torres-Martos, Aiman Tariq Baig, Concepción M. Aguilera, Francisco Javier Ruiz-Ojeda

**Affiliations:** 1 Department of Biochemistry and Molecular Biology II, School of Pharmacy, University of Granada, 18071 Granada, Spain; davidizquierdo@correo.ugr.es (D.I.); caguiler@ugr.es (C.M.A.); 2 Instituto de Investigación Biosanitaria IBS.GRANADA, Complejo Hospitalario Universitario de Granada, 18014 Granada, Spain; alvarotorresmartos@gmail.com; 3 Children’s Hospital of Eastern Ontario Research Institute, Ottawa, ON K1H 8L1, Canada; abaig034@uottawa.ca; 4 Department of Cellular and Molecular Medicine, Faculty of Medicine, University of Ottawa, Ottawa, ON K1H 85M, Canada; 5 Center of Biomedical Research, Institute of Nutrition and Food Technology “José Mataix”, University of Granada, Avda. del Conocimiento s/n., 18016 Granada, Spain; 6 CIBEROBN (CIBER Physiopathology of Obesity and Nutrition), Instituto de Salud Carlos III, 28029 Madrid, Spain; 7 RG Adipocytes and Metabolism, Institute for Diabetes and Obesity, Helmholtz Diabetes Center at Helmholtz, Center Munich, Neuherberg, 85764 Munich, Germany

**Keywords:** epigenetics, exercise, physical activity, skeletal muscle, metabolism

## Abstract

Exercise and physical activity induces physiological responses in organisms, and adaptations in skeletal muscle, which is beneficial for maintaining health and preventing and/or treating most chronic diseases. These adaptations are mainly instigated by transcriptional responses that ensue in reaction to each individual exercise, either resistance or endurance. Consequently, changes in key metabolic, regulatory, and myogenic genes in skeletal muscle occur as both an early and late response to exercise, and these epigenetic modifications, which are influenced by environmental and genetic factors, trigger those alterations in the transcriptional responses. DNA methylation and histone modifications are the most significant epigenetic changes described in gene transcription, linked to the skeletal muscle transcriptional response to exercise, and mediating the exercise adaptations. Nevertheless, other alterations in the epigenetics markers, such as epitranscriptomics, modifications mediated by miRNAs, and lactylation as a novel epigenetic modification, are emerging as key events for gene transcription. Here, we provide an overview and update of the impact of exercise on epigenetic modifications, including the well-described DNA methylations and histone modifications, and the emerging modifications in the skeletal muscle. In addition, we describe the effects of exercise on epigenetic markers in other metabolic tissues; also, we provide information about how systemic metabolism or its metabolites influence epigenetic modifications in the skeletal muscle.

## 1. Introduction

Physical activity is defined as every physical action using skeletal muscles that produces an energy expenditure in daily time and can be classified into sports, occupational, household, conditioning, or other activities [1]. Exercise is a subsection of physical activity that is structured, premeditated, and cyclic and has as a final or an intermediate objective the progress or physical fitness maintenance related to both skills- or health-related aspects [2,3]. Physical activity and exercise are essential to maintain a healthy metabolism and burn more calories per day. In short, exercise is not just significant for general health, it is crucial to the molecular memory of who we are, simply because the lack of exercise increases the risk of obesity and type 2 diabetes, as well as developing diseases linked to brain function, such as dementia, psychiatric disorders, and even violent behavior [4,5]. Humans today may have a higher metabolic capacity than our ancestors did, measured by our capacity to take up and use oxygen (VO_2_max) [5]. A lack of exercise might have multiple, long-term damaging effects, in particular when coupled with a poor diet. Consequently, nowadays, the prevalence of chronic diseases associated with a sedentary lifestyle and poor diet has increased alarmingly [6].

Exercise is utilized to maintain or restore whole-body homeostasis and human locomotion, and it can cause several metabolic adaptations in the skeletal muscle in order to improve the performance of the body [7]. Therefore, a better understanding of the underlying mechanisms responsible for these adaptations will help to improve training guidelines [8]. Skeletal muscle is a plastic tissue capable of adapting rapidly in response to changes in metabolic homeostasis induced by exercise. In the physiology of exercise, two types of physical exercise are mainly differentiated: endurance training, which is characterized by using low and repeated loads, where the work of the cardiorespiratory system predominates; the term endurance training generally refers to training the aerobic system as opposed to the anaerobic system and strength or resistance training, which uses higher loads at low repetition exercise bouts and is focused more on working the neuromuscular system [9]. It also uses resistance to muscular contraction to build the strength, anaerobic endurance and size of skeletal muscles [10]. Most activities combine endurance and strength and this type of training has been termed concurrent exercise [11].

During exercise, both in endurance and resistance training, a series of acute responses occur in almost every system and tissue in the body. First, the motor cortex recruits the motor units of the target muscle to produce movement. Depending on the type of movement you want to achieve, different fibers will be activated. There are several types of fibers in our muscles; type I fibers are more resistant to fatigue and have a predominantly oxidative metabolism, whereas type IIx fibers are more glycolytic, fatigued and fast-twitch, and type IIa fibers are a combination of both [12]. The cardiovascular, respiratory, and hormonal systems and various metabolic processes are also activated in parallel, leading to a complete disruption of homeostasis [13]. The physiological responses prompt the activation of several kinases, including adenosine monophosphate (AMP)-activated protein kinase (AMPK), Protein kinase A (PKA), calcium/calmodulin-dependent protein kinase (CaMK), mitogen-activated protein kinase (MAPK), Protein kinase C (PKC) and Mammalian target of rapamycin (mTOR) [14]. The energy-sensing kinase AMPK, which is regulated by cellular energy deficit [15,16], plays an important role in the beneficial effects of exercise on whole-body metabolic homeostasis. Indeed, muscle-specific AMPK knockout mouse models exhibit a pivotal role for AMPK in the metabolic adaptation of muscle during exercise. Nonetheless, numerous reports have suggested the previously characterized metabolic disturbances induced with exercise are not completely dependent on AMPK.

Overall, AMPK activation through physical exercise improves mitochondrial biogenesis through the regulation of peroxisome proliferator-activated receptor gamma coactivator 1-alpha (PGC-1α), which promotes the expression of mitochondrial genes encoded in mitochondrial and nuclear DNA [17]. CaMK-II is another highly conserved studied protein, which is dependent on the intensity of exercise and whose activation promotes the activation of PGC-1α and glucose transporter 4 (GLUT-4) [18]. Moreover, CaMK-II promotes lipid uptake and oxidation and skeletal muscle plasticity by disrupting members of myocyte Enhancer Factor 2 histone deacetylase (MEF2-HDAC) complexes and stimulating HDAC nuclear export; it also triggers regulation of important transcription factors, such as the cyclic AMP response element-binding protein (CREB), MEF2, and HDACs in skeletal muscle [19]. Finally, it is worth mentioning that there are several differences in molecular responses to exercise between endurance and resistance exercise. Generally, resistance training increases the activation of the phosphoinositide 3-kinases Protein kinase B–mTOR (PI3k-Akt-mTOR) signaling cascade to regulate the rate of protein synthesis and/or degradation and consequently, muscle hypertrophy. Nevertheless, endurance training activates the AMPK-MAPK-PGC-1α signaling cascades, ultimately leading to increased mitochondrial biogenesis [13] and metabolic adaptations such as fast-to-slow muscle fiber transition, as well as angiogenesis.

In recent years, emerging evidence suggests that epigenetic modifications may mediate the intergenerational transmission of exercise effects on physiology. Epigenetics is a concept conceived by Conrad Waddington in 1940. Since then, the attention on this field has increased enormously, and herein, the definition of epigenetics has progressed toward changes in transcriptional expression and/or activity without variation in DNA sequence [20]. These changes are of vital importance in fundamental biology, regulating such processes as genomic imprinting and X chromosome inactivation, as well as being the most reliable molecular method of human biological age prediction, collectively underlying its importance and association with development, disease, and aging [21,22]. DNA methylation and histone modifications have been the most studied epigenetic events. Nevertheless, other potential epigenetic modifications such as those mediated by microRNAs (miRNAs) may alter gene expression via post-transcriptional modulation and may influence translational events. Furthermore, the identification of circulating miRNAs provided the possibility that they are involved in cell–cell and tissue–tissue communication [23]. Overall, the interactions between multiple epigenetic modifications and their regulation by metabolism during exercise are complex, and the comprehensive understanding of these adaptations needs to be further investigated.

In the present review, we provide an overview and update of the impact of exercise on the epigenome, including DNA methylation, histone modifications, miRNAs, epitranscriptomics, and novel mechanisms, such as lactylation in the skeletal muscle. In addition, we highlight the effect of exercise on epigenetic changes in other metabolic tissues such as adipose tissue, liver, pancreas, and brain, and we summarize the main effects of metabolism and its metabolites on epigenetic modifications in the skeletal muscle.

## 2. Methods

A comprehensive search of the relevant literature was performed in the electronic database MEDLINE through PubMed (U.S. National Library of Medicine and the NIH). The following phrases were included in the search of the literature without time restrictions: ((exercise[Title/Abstract]) OR (aerobic exercise[MeSH Terms]) OR (activity, physical[MeSH Terms])) AND (humans) AND ((epigenetic[MeSH Terms]) OR (epigenetic[Title/Abstract])) AND (muscle, skeletal[MeSH Terms]). In addition, in order to revise the evidence related to epigenetic and effects on systemic metabolism, we included the following phrases in the search: “epigenomics”, ”exercise”, “muscle, skeletal”, “DNA methylation”, “histone code”, “microRNAs”, “adipose tissue”, “liver”, “pancreas” and “brain”. Finally, previous original articles and reviews focusing on epigenetics and skeletal muscle in the context of exercise and physical activity were carefully examined.

## 3. Physical Activity and Exercise and the Epigenetic Changes in Skeletal Muscle

Epigenetics constitutes an important level of regulation in the expression of genes without changes in the DNA sequence [24]. The mechanisms involved in the regulation of gene expression are heritable, and the modifications that occur in the nucleotide sequence are reversible [25,26]. Each cell type possesses a distinct innate epigenetic signature or hallmark called the epigenome, which controls the conformation of the DNA strand in two distinct states, euchromatin and heterochromatin, by which access to the DNA of the transcription machinery is controlled [27]. It has been shown that epigenetic modifications can be influenced in a tissue-specific manner by environmental stimuli such as diet, smoking, or exercise [28]. In this context, epigenetic changes can be considered as the intersection between genetics and the environment (nature vs. nurture) **[29]**. Some epigenetic changes may play a key role in skeletal muscle, a malleable organ that responds to training sessions by inducing the expression of genes involved in structural, metabolic, and functional adaptations leading to transient changes [30,31]. This suggests that epigenetic mechanisms are not only restricted to early developmental stages in humans, but also dynamic controllers of genomic plasticity in response to environmental factors [32]. Three major epigenetic modifications regulate gene expression, DNA methylation, histone modifications, and miRNA activity [31,32,33].

### 3.1. Skeletal Muscle Physiology and Adaptations to Exercise

The skeletal muscle is composed of 75% water, 20% protein, and 5% other compounds [34]. Total muscle mass depends on the balance between synthesis and degradation of proteins, which are processes sensitive to the nutritional state and hormonal balance of the individual, the physical activity or exercise performed, and the presence of any type of injury or disease [35,36]. The heterogeneity of skeletal muscle is a property of all muscles, which allows them to maintain their function in response to a wide range of demands [37]. Thus, the presence of fibers with different properties in the same muscle is the result of an adaptation to different activity patterns imposed by motor neurons, which allows the muscle to participate in activities with various metabolic and mechanical demands [38].

Skeletal muscle fibers are commonly classified as type I (slow contraction fiber, predominant oxidative metabolism, and fatigue resistance), type IIA (fast contraction fiber, predominant oxidative metabolism), and type IIx (a fiber that presents the fastest contraction pattern, with predominant glycolytic metabolism and a high degree of fatigue in sustained activities) [36]. During endurance exercise, large muscle groups are activated at an intensity that requires high efficiency in oxygen transport and release [39,40]. This process drives an increase in capillary supply to facilitate O_2_ uptake and transport, rising in the number and size of mitochondria, reinforcement of fat, and glycogen storage [41]. Furthermore, endurance exercise increases the concentration of oxidative enzymes of the Krebs cycle necessary for the aerobic production of ATP and facilitates greater development of the sarcoplasmic Ca^2+^ reticulum, deregulation of oxygen transport proteins, and improvement of metabolic capacity by increasing the synthesis of mitochondrial proteins without changes in the synthesis of myofibrillar proteins [36,42]. In contrast, resistance exercise increments the ability to generate force due in part to muscle hypertrophy that occurs as a result of the activation and fusion of satellite cells [43]. Moreover, higher myonuclei are produced and this results in increased protein synthesis and a number of myofilaments, myofibrils, and sarcomeres, increasing the individual muscle fibers’ size [9,44].

Several external factors influence muscle hypertrophy induced by resistance exercises, such as the intensity and load used during exercise or the availability of macronutrients, all interacting with the individual’s genotype to determine muscle growth [45,46]. Muscle hypertrophy is the most recognized adaptation of resistance exercise, but other adaptations occur to support the biochemical, physical, and metabolic requirements of muscle growth [47]. The recognized hypertrophy results from complex intra- and extra-muscular communication to coordinate muscle growth [48]. Thus, a transient alteration occurs in the muscle after a training session, which results in a new stable state of the skeletal muscle [49]. Overall, skeletal muscle contraction during exercise raises force production and energy demand, with simultaneous activation of metabolic pathways and the physiological adaptation for oxygen and substrate supply, carbon dioxide and heat removal, fluid balance, and homeostatic regulation [7,50].

### 3.2. Epigenetics and Physical Activity/Exercise in Skeletal Muscle

Existing knowledge of the molecular mechanisms involved in the adaptations conferred by exercise suggests that repeated and transient increases in the expression of exercise-responsive genes in skeletal muscle confer such adaptations over time, contributing to the positive effects of physical activity [51]. The regulator and adaptableness of biological processes involve post-synthesis chemical variation of three classes of fundamental macromolecules: DNA, RNA, and proteins. One of the most profuse variations is DNA methylation, which is extensive through all kingdoms of life and includes an alkylation reaction whereby a methyl group switches a hydrogen atom [27]. DNA methylation is one of the epigenetic mechanisms with the greatest influence on gene activity [52].

#### 3.2.1. DNA Methylation

DNA methylation is one of the epigenetic mechanisms with the greatest influence on gene activity [52]. Vertebrate DNA can be covalently modified by methylation of cytosines present in the 5′CpG3′ dinucleotide sequence. CpG is the abbreviation used to refer to the joining of the two nitrogenous bases, cytosine and guanine, separated by a phosphate that binds them to the DNA strand [53]. This process is catalyzed by a family of DNA methyltransferases (DNMT) that transfer a methyl group from S-adenyl methionine (SAM) to carbon 5 of the cytosine residue to form 5mC (5-methylcytosine) [54,55].

Most DNA methylation occurs on cytosines found in the known CpG as islands. The majority of gene promoters (approximately 70%) reside in CpG islands [52]. These sequences are highly conserved, and their localization and conservation throughout evolution imply that they are functionally important regions. It appears that CpG islands have been conserved throughout evolution to promote gene expression by regulating chromatin structure and DNA binding of transcription factors [56,57].

DNMTs are enzymes that establish, recognize, and remove DNA methylation, they and are divided into three groups according to their function. DNMT writers that catalyze the addition of a methyl group to the cytosine residue (Dnmt1, Dnmt3a, and Dnmt3b); DNMT readers, which are enzymes that recognize the methyl group and bind to it to influence gene expression; DNA methyltransferases erasers, enzymes responsible for modifying and removing the 5mC methyl group to reverse DNA methylation [52,58].

Commonly, DNA methylation results in the stable silencing of gene expression by repressing transcription [59]. Exercise generally results in DNA hypomethylation in key skeletal muscle genes, representing an early response that mediates skeletal muscle adaptations to exercise [41]. Thus, muscle contraction through physical exercise leads to adaptive responses that improve metabolic efficiency, oxidative capacity, and contractile activity by altering gene expression profiles and protein levels [60]. One hypothesis explaining how exercise triggers DNA methylation suggests that during muscle contraction several processes occur such as cytoplasmic Ca^2+^ release from, and subsequent reabsorption by, the sarcoplasmic reticulum and ATP consumption by myosin movement that alter the AMP–ATP ratio, 1 in AMPK activation [61]. There is also an increase in oxidative metabolism for the production of ATP necessary for muscle contraction [62]. This generates reactive oxygen species (ROS) that induce DNA to trigger a genomic response. ROS are modulated by members of carbon metabolism such as S-adenosyl methionine (SAM), which serve as donors of methyl groups used in DNA methylation [63]. Consequently, modulating the availability of methyl donors is how oxidative stress, along with calcium, could be the triggers that control exercise-induced methylation [32,64].

An example is found in the genes coding for the proinflammatory cytokines as interleukin 6 (IL-6) and tumor necrosis factor α (TNF-α). These are hypomethylated in skeletal muscle of sedentary people at rest when compared with people who practice strength training (sedentary people present greater expression of these genes so that their state of inflammation is greater) [65]. IL-6 protein levels normally increase when performing an intense exercise session, these levels are also higher in sedentary people than in people who practice exercise. This indicates a chronic adaptation in people who perform exercise that creates a methylation profile more prepared for recovery after physical activity by decreasing the basal expression of proinflammatory mediators [66].

*PGC1-α* is a key regulatory gene for mitochondrial biogenesis, fatty acid oxidation, and skeletal muscle sensitivity to insulin. The *PGC-1α* gene is hypomethylated after an intense exercise session [32,67,68]. Barrès et al. performed biopsies of the *vastus lateralis*, in which they found a different methylation state of the promoter of this gene, specifically, 10% less methylated compared to the resting state. Hypomethylation levels of *PCG-1α* correlate with increased mRNA levels three hours after endurance exercise, this affirms that changes in methylation are involved in the activation of transcription [60]. This gene and its control by epigenetic regulations are involved in numerous diseases and physiological conditions: regular sports practice in pregnant women prevents PGC-1α hypermethylation induced by high-fat diets in offspring, and increases PGC-1α levels, thereby ameliorating age-associated metabolic dysfunction. These data suggest that DNA methylation in PGC-1α may be related to metabolic memory in endurance exercise [69]. Moreover, 4 days of inactivity increases expression of genes in muscle associated with insulin resistance and increased DNA methylation in PGC-1α in endurance exercise [70].

Training can alter the DNA methylation status of multiple genes in a dose-dependent manner. There is an indirect correlation between DNA methylation levels and the resulting levels of mRNA expression of several genes, but not all genes are studied in the skeletal muscle adaptive response to exercise [31,60,67]. In this context, changes in DNA methylation have been observed both immediately after an acute exercise session and chronically after a training program of several weeks or months. The magnitude of these changes is less after following a training program than after a single intense exercise session, indicating that changes in DNA methylation in response to exercise are a dynamic process that is activated early in gene expression. Nevertheless, residual changes in methylation are also maintained after the training stimulus disappears, indicating that they accumulate over multiple exercise sessions. In addition, it has been observed that the basal levels of methylation prior to the training program (levels typical of the untrained state) are not restored in the short term [31,32]. Therefore, several genes involved in the adaptive response of skeletal muscle to exercise have been studied in the published literature (Table 1).

TFAM (Mitochondrial Transcription Factor A) is a mitochondrial DNA (mtDNA) regulatory protein that protects from ROS and degradation while increasing mitochondrial function [71]. *TFAM* gene promoters are hypomethylated after just one exercise session and are maintained 3 h later. The mRNA levels also increased, but they do so immediately after the end of either endurance or resistance exercise [32,67]. The same occurs with peroxisome proliferator-activated receptor-gamma (*PPAR-γ*) mRNA levels, but this gene shows delayed hypomethylation, indicating that DNA modifications may depend on exercise intensity (endurance or resistance) [31].

*PDK4* (Pyruvate dehydrogenase kinase 4) is a key gene in skeletal muscle metabolism and its expression is associated with hyperglycemia and is increased after either high-intensity exercise for a short period of time or after prolonged low-intensity exercise, and remains elevated as a consequence of chronic exercise. Its promoter is hypomethylated just after exercise, but transcription does not increase until 3 h later, as is the case with PGC-1α in either endurance or resistance exercise [67]. The methylation levels of *MEF2* (myocyte Specific Enhancer Factor 2) and *CS* (Citrate Synthase) decrease in response to exercise, but there are no significant changes in mRNA expression in endurance exercise [60].

**Table 1:** Effects of endurance and resistance training on DNA methylation in skeletal muscle.

Endurance Exercise							
Reference	Sample Size	Age and Sex	Participant Profiles	Exercise Doses	Biopsy Times	Technology	Methylation Changes and Gene Expression
Barres et al., (2012) [60]	n = 14	25 ± 1 years, men and women	Sedentary	Acute session, Intensity: 80% VO_2_, Volume: until 1.674 kJ	Before, after and 3 h post-exercise	Pyrosequencing	Hypomethylation of *PGC-1α, TFAM, MEF2A Y PDK4* after exercise. Hypomethylation of *PPAR-d* 3 h post exercise
Bajpeyi et al., (2017) [72]	n = 11	24 ± 1 years, men	High and low responders	Acute session, Intensity: 50% VO_2_, Volume: until 650 kcal	Before and after exercise	Pyrosequencing	Hypomethylation of PGC1α and higher mRNA levels in responders to exercise
Lane et al., (2015) [73]	n = 7	29 ± 5 years, men	Cyclists completing two trials receiving isoenergetic diets differing in the timing of ingestion	Acute session, Intensity: 50% VO_2_, Duration: 120 min	Before and after exercise	Pyrosequencing	Hypermethylation of *COX411* y *FABP3* 4 h after the training session. DNA methylation of PPARs increased only in the fasting group
Nitert et al., (2012) [74]	n = 28	37.5 ± 5.2 years, men	Individuals with/without familiar diabetes history	6 months of endurance exercise and spinning (2–3 sessions per week, 1 h)	Before and after exercise	MeDIP-Chip	Hypomethylation of *RUNX1, MEF2A, THADA y NDUFC2*
Alibegovic et al., (2010) [70]	n = 20	25 ± 1 years, men	Healthy Caucasian without Type 2 diabetes antecedents	4 weeks, 6 days per week, Volume: 30 min, Intensity: 70% VO_2_ max	Before and after exercise	EZ DNA Methylation kit	Hypermethylation of *PPARGC1A* after 8 bed rest. Hypomethylation of PPARGC1A after a training program
Robinson et al., (2017) [75]	n = 34	29 ± 5 years, men and women	Adults	12 weeks, 3 times per week, 4 × 4, 90% VO_2,_ 3 min active rest, 3 days of treadmill, walking (45 min 70%)	Before and after exercise	450 K array (Illumina)	Shifts in the DNA methylation less than 10%
Lindholm et al., (2014) [76]	n = 23	27 ± 0.8, men and women	Young people (without practicing exercise)	3 months (resistance to 1 leg), 4 sessions, 45 min	Resting, before and after	450 K array (Illumina)	Changes in DNA methylation in 5000 sites and different gene expression in 4000 genes.
Turner et al., (2020) [77]	n = 30	27 ± 4.4 years, men	Young adults	1-6 sessions per week for 6 months	-	850 K Array (Illumina)	Hypomethylation of *HOXB1 y HOXA3*.
Sailani et al., (2019) [78]	n = 8	62.1 ± 1.3 years, men	Healthy individuals, (performing regular exercise or remained sedentary their entire lives)	More than 3 times per week	-	850 K Array (Illumina)	Hypomethylation in 714 promoters of the physically active than inactive men. Promoters for genes encoding critical insulin-responsive enzymes in glycogen metabolism, glycolysis and TCA cycle were hypomethylated in active relative to inactive men.
Rowlands et al., (2014) [79]	n = 8	49 ± 5 years, Men and women	Individuals with type 2 diabetes and obesity	16 weeks, 3 days per week, 40–60 min per session	Before and after exercise	450 K array (Illumina)	Hypermethylation in *NRF1 y SLC27A4*
Stephens et al., (2018) [80]	n = 17	50.7 ± 1.9 years, women	Individuals with type 2 diabetes	10 weeks, 4 days per week, Progressive intensity	Before and after exercise	450 K array (Illumina)	Hypermethylation in responders compared to non-responders.
Maasar et al., (2021) [68]	n = 5	26 ± 2 years, men	Sport team members	(1) change of direction (COD) versus; (2) straight line (ST), running exercise. Wash-out period of at least 2 weeks between trials.	Before (30 min) and 24 h after exercise	850 K Array (Illumina)	Hypomethylation after 30 min, mainly in *AMPK, MAPK*, protein binding, insulin, and axon guidance pathways. Hypermethylation of VEGFA, PPARGC1A, NR4A3
**Resistance Exercise**							
**Reference**	**Sample Size**	**Age and Sex**	**Participants Profile**	**Exercise Doses**	**Biopsies Time**	**Technology**	**Methylation Changes** **and Gene Expression**
Rowlands et al., (2014) [79]	n = 9	49 ± 5 years, men and women	Type 2 diabetes and obesity	16 weeks, 3 days per week, Participants were randomized into endurance or resistance exercise groups comprising supervised progressive-loading exercise sessions 3 ×/week on non-consecutive days	Before and after exercise	450 K Array (Illumina)	Hypomethylation of 409 CpGs sites and hypermethylation of 146 CpGs sites.
Seaborne et al., (2018) [81]	n = 8	27.6 ± 2.4 years, men	Adults (non-trained)	An acute bout of resistance exercise (acute RE), followed by 7 weeks (3d/week) of resistance exercise (loading), 7 weeks of exercise cessation (unloading) and a further period of 7 weeks (3d/week) resistance exercise (re-loading).	Before the first training session, after acute exercise, after a period of 7 weeks of resistance exercise (loading), exercise cessation (unloading) and a subsequent second period of 7 weeks resistance exercise (reloading).	DNA microarray	Hypomethylation of *AXIN1, GRIK2, CAMK-IV, TRAF1, UBR5, RPL35a, HEG1, PLA2G16 y SETD3*
Bagley et al., (2020) [66]	n = 11 and n = 8	26.2 ± 0.1 years, and 22.9 ±1.1 years	Trained vs sedentary young individuals	3 × 10 repeats, 70% RM, press and leg extension	Before and 4 h after exercise	PCR	Global DNA hypomethylation in trained individuals compared to sedentary. Hypermethylation of GPAM y SREBF2 in trained individuals and hypomethylation of SREBF2 in sedentary individuals. No changes in DNA methylation of genes are associated with hypertrophy and inflammation.

Abbreviations. AMPK: AMP-activated protein kinase; AXIN1: CAMK-IV: calcium/calmodulin-dependent protein kinase type IV; GPAM: mitochondrial glycerol-3-phosphate acyltransferase 1; GRIK2: Ionotropic glutamate kainate receptor type 2 subunit; HEG1: Cardiac development protein with EGF 1-like domains; HOXA3: HOXB1: MAPK: mitogen-activated protein kinases; MEF2A: myocyte-specific enhancer factor 2a; NDUFC2: C2 subunit of NADH dehydrogenase; NR4A3: nuclear receptor 4A3; NRF1: nuclear respiratory factor 1; PDK4: pyruvate dehydrogenase kinase 3; PGC-1α/PPARGC1A: peroxisome proliferator-activated receptor gamma 1-alpha coactivator; PLA2G16: PPAR: Peroxisomal Proliferator Activated Receptors; RPL35a: RUNX1: redness-related transcription factor 1; SETD3: SET domain containing 3; SLC27A4: solute carrier family 27 member 4; SREBF2: sterol regulatory element binding protein 2; TFAM: mitochondrial transcription factor A, THADA: TRAF1: TNF receptor associated factor 1; UBR5: E3 ubiquitin-protein ligase UBR5; THADA, associated thyroid adenoma is a protein.

In a recent study, Figueiredo et al. (2021) revealed the impact of genetic and epigenetic mechanisms on skeletal muscle ribosome biogenesis in humans during both resistance and endurance exercise by evaluating the time-course of ribosome biogenesis and rRNA transcription regulatory factor responses. They showed that ribosome biogenesis and *MYC* transcription are associated principally with resistance but not endurance exercise, signifying preferential up-regulation during hypertrophic processes. With resistance exercise, ribosome biogenesis was associated with rDNA gene dosage, as well as epigenetic changes in enhancer and non-canonical MYC-associated areas in rDNA, but not the promoter. Overall, this study provides for the first-time new insights related to the regulation of ribosome biogenesis during exercise in humans. It seems that resistance exercise is more disposed to induce ribosome biogenesis than endurance exercise, and epigenetic mechanisms are critical regulators of this process [82,83]. In addition to that, they found that endurance exercise increases AMPK phosphorylation at the Thr172 site after 30 min of exercise in skeletal muscle biopsies from *vastus lateralis*. Furthermore, they showed that resistance exercise activated mTORC1 signaling by increasing the ribosomal protein S6 kinase (p70S6K) at Thr389 and ribosomal protein S6 (rpS6) at Ser240/244, confirming previous results observed in the literature [84].

A recent study revealed that resistance exercise training affects mtDNA methylation patterns in skeletal muscle, as 63% (159/254) of the GpG sites demonstrated reduced methylation. Some of the mtDNA sites presented a more “youthful” signature in older males after resistance training compared to younger males. Furthermore, enhanced expression of mitochondrial H-and L-strand genes and complex III/IV protein levels were also observed in this study [85].

#### 3.2.2. Histone Modifications

Histones contain two domains, a central region (long alpha-helix flanked by two short alpha-helices) folded and interacting with DNA, and an N-terminal domain of 15–30 residues called “histone tails”. These histone tails emerge from the central region of the nucleosome and undergo various post-translational modifications that affect chromatin structure and function. Epigenetic modifications are responsible for transforming the packaging of DNA and whether or not it can be utilized [86].

The most important histone post-translational modifications are acetylation, methylation, and phosphorylation. This wide variety of modifications and the combination among them provides a great potential for functional responses since they are dynamic changes that vary rapidly in response to cellular stimuli [87].

The “histone code” is the set of modifications undergone by histones and read by effector proteins that initiate biological responses such as activation or repression of transcription [88,89,90]. Some modifications are associated with activation and others with gene repression, although the effects seem to depend on the context in which they occur. DNA in euchromatin has greater functional flexibility, genes can be activated or remain inactive, and DNA can be unpacked for replication or repair. Transcriptionally active euchromatin has high levels of acetylation while transcriptionally inactive heterochromatin has low levels of acetylation, phosphorylation, and methylation [91,92]. On the other hand, inactive heterochromatin is associated with low levels of acetylation and high levels of methylation of some histone residues [93].

Exercise has a profound effect on the distribution of the histone markers. Many studies have found increased levels of methylation in H3K4 (histone H3 lysine 4), acetylation in H3K27 (histone H3 lysine 27), and phosphorylation in serine in H3 in skeletal muscle. The levels of the H3K27ac mark are a prominent marker of enhancer activity after 6 weeks of endurance training (Table 2) [94].

##### Acetylation

Histone acetylation is a transient enzymatic process that is the most common histone post-translational modification. The acetyl group of acetyl-CoA is transferred to a lysine residue of the histone tails [97]. Changes in the positive charges generated a DNA molecule more exposed and accessible to regulatory proteins and associating acetylation with gene activation [98,99,100].

DNA acetylation is mediated by histone acetyltransferases (HATs) that add an acetyl group to the histone, and histone deacetylases (HDACs) that remove it. The relative levels of histone acetylation are determined by the opposing enzymatic activities of HATs and HDACs [33]. Exercise is associated with the acetylation of several lysine residues in human skeletal muscle histones, so that physical activity correlates with chromatin decompaction and activation of transcription of certain exercise-responsive genes [101,102]. The practice of intense strength exercise produces an increase in histone H3 acetylation [103].

##### Methylation

Methylation takes place at the lysine and arginine residues of histones H3 and H4, to which a methyl group is added [104]. Histone methyltransferases (HMT) are responsible for catalyzing this reaction, using SAM as a substrate to transfer a methyl group to the lysines [105]. Methylated lysines and arginines can activate or repress gene transcription depending on the proteins they recruit to chromatin [97].

Lysines can be mono-, di-, or trimethylated (me1, me2, and me3 respectively) which will result in different responses. In general, methylation of H3K4, H3K36, and H3K79 is associated with epigenetic marks that activate gene transcription, whereas methylation of H3K9, H3K27, and H4K20 is associated with chromatin condensation and repression of gene expression [106]. H3K4 methylation is highly abundant in promoter regions and transcriptional start sites and increases with physical exercise [107].

##### Phosphorylation

Phosphorylation occurs at the serine and tyrosine residues of histones [108]. Exercise causes increased levels of H3 serine phosphorylation in skeletal muscle [96]. Thus, certain signaling pathways including AMPK, MAPK, PKA, PKC, and CaMK-II are important for phosphorylation-dependent signaling during exercise in skeletal muscle [14].

Many studies relate these pathways to histone modifications. Both AMPK and CaMK-II directly phosphorylate H3 [109,110]. The available theory suggests that H3 phosphorylation is necessary prior to acetylation, i.e., there is a step-by-step control of chromatin decompaction and mechanisms necessary for the initiation of transcription [111].

##### Lactylation

Lactate is a cell marker of metabolic state, which results in epigenetics and transcriptomics changes in the cell. Furthermore, lactate inhibits the HDAC activity and thus increases gene expression boosting lactate availability during exercise [112].

Lysine lactylation is an epigenetic modification that occurs in the presence of elevated levels of lactate [113]. During exercise, lactylation appears in promoters of coding genes; this epigenetic code is associated with changes in transcriptional patterns [22]. Some authors have found evidence that lactylation mechanisms are based on Lactyl-CoA, an epigenetic “writer” in macrophage cell models. Moreover, they discovered that lysine lactylation is involved in the up-regulation of homeostatic genes. It is suggested that lactate and lactylation could play a communication role between cells and tissue inducing adaptive responses during and after exercise [114]. However, the mechanisms and metabolic implications of lactylation in skeletal muscle remain unclear and for that, future in-depth research on lactylation would be necessary.

#### 3.2.3. Micro-RNAs

Micro-RNAs are small non-coding RNAs that generally repress the expression of several genes (from one hundred to a thousand) at a post-transcriptional level. The presence of micro-RNAs is usually due to non-homeostatic conditions and for that reason, they are relevant in the study of exercise physiology [30]. Micro-RNAs arising from skeletal or cardiac muscle are called myomiRs. Currently, only seven myomiRs related to skeletal muscle have been identified: miR-1, miR-133a, miR-133b, miR-206 (expressed only in skeletal muscle), miR-208b, miR-486, and miR-499, and their expression levels depend on the type and length of the exercise [31]. In general, the myomiRs’ functions are to control the biogenesis, regeneration, and maintenance of the skeletal muscle tissue [115]. The evidence suggests that exercise increases the expression of proteins, which are implicated in micro-RNAs maturation, for example, Drosha, Dicer, and Exportin-5, and raises expression levels of myomiRs. For that reason, it can be assumed that exercise and micro-RNAs have a much-linked relationship [116].

MyomiRs control several post-training processes regulating the expression of different genes. Firstly, miR-133a promotes the myoblast proliferation; secondly, miR-1, miR133a, miR133b, miR-206, and miR-486 stimulate the myoblast differentiation (miR-1 and miR-206 down-regulate HDAC4). Then, miR-133a, miR-133b, and mir486 result in muscle cell fusion, and lastly, miR-1, miR-133a, and miR-206 encourage muscle regeneration. In addition, myomiRs are involved in other processes such as muscle fiber shift and muscle growth, as in the case of miR-133a, miR-208b, and miR-499 [117]. It is worth pointing out that miR-1 and miR-206 have been proposed as biomarkers of endurance [118]. Table 3 summarize the effects of endurance and resistance exercise on mi-RNAs generation in skeletal muscle. 

Mainly, one unique micro-RNA can interact with many target mRNAs and one unique mRNA can interact with several micro-RNAs at the same time. This fact makes it difficult to understand molecular pathways in which micro-RNAs are implicated [115]. Some authors have tried to provide more complete information about the myomiRs’ and other micro-RNAs’ implications in these complex pathways related to myogenesis and muscle-skeletal differentiation.

**Table 3:** Effects of endurance and resistance training on mi-RNAs generation in skeletal muscle.

Endurance Exercise							
Reference	Sample Size	Age and Sex	Participant Profiles	Exercise Doses	Biopsy Times	Technology	Epigenetic Changes and Gene Expression
Russel et al., (2013) [116]	n = 9	23 ± 5 years, men	Healthy people (less than 2 h of exercise per week)	Acute: 60 min 70%VO_2_max, Chronic (10 days), Progression: from 45 a 90 min to 75%VO_2_, 4 days of HIIT: 6 × 5 min (90–100% VO_2_), 2 min of resting	Before and after intervention	TaqMan qRT-PCR	Acute: up-regulation of miR-1, −133a, 133b, −181 and down-regulation of miR-9, −23a, −23b, y −31. Chronic: up-regulation of miR-29b and down-regulation of miR-31
Keller et al., (2011) [119]	n = 8	29 ± 6 years, men	Sedentary healthy individuals	4 days/week, 70% VO_2_max, 45 min	Before and after intervention	TaqMan RT-PCR	Lower expression of miRNAs (14 vs 7), Lower levels of miR-1, miR-133, miR-101 y miR-455.
Nielsen et al., (2010) [120]	n = 10	30.5 ± 5.5 years	Trained individuals	Acute: 60 min, 65% Pmax, Chronic (12 weeks), 5 days per week, 55–91% Pmax, 60–150 min	Before, 1 h before and 3 h after intervention	TaqMan RT-PCR	Acute: Higher expression of miR-1 and −133a, Chronic: all miRNAs were lower and restored after 2 weeks of intervention
Fyfe, J.J. et al., (2016) [121]	n = 8	27± 4 years, Men	Active young individuals	2 × 10 min, 1 min rest, 120% lactic umbral	Before, 1 h before and 3 h after intervention	TaqMan RT-PCR	Lower expression of miR-133a, miR-378 y miR-486
Margolis, L.M. et al., (2017) [122]	n = 25	18–40 years, Men and women		90 min, 2.2 ± 0.1 L/min,	Before and 3 h after intervention	TaqMan RT-PCR	Lower expression of myomiR in the highest loaded group (miR-1-3p, miR-206, miR-208a-5p, y miR-499), Higher expression of myomiR in the endurance group
**Resistance Exercise**							
**Reference**	**Sample Size**	**Age and Sex**	**Participants Profile**	**Exercise Doses**	**Biopsies Time**	**Technology**	**Epigenetic Changes and Gene Expression**
Davidsen et al., (2011) [123]	n = 56	18–30 years, men	Active individuals	12 weeks 5 days/week 60 min per session 20 sets by muscle group	Before and after intervention	TaqMan RT-PCR	17 miRNAs were detected, and miR-78, miR-29a, miR-26a, and miR-451 were lower in the low-responders. miR-451 was up-regulated.
Rivas et al., (2014) [124]	n = 8	22 ± 1 years, 74 ± 2 years, men	Adults, Young and old people	3 series of 10 repetitions, 80% Maximun repetition, 2 types of exercises	Before and 6 h after intervention	PCR-Array	17 miRNAs were differentially expressed in young people and no changes were found in old individuals. Only miR-423-5p was up-regulated in both young and old individuals.
Ogasawara et al., (2016) [125]	n = 18	21.4 ± 1.1 years, men	Healthy and trained (resistance) individuals	12 weeks: 10 repetitions at 70% of 1 repetition maximum (RM) for 3 sets with 2 min rest intervals. 3 days per week on alternative days for 6 week.	Before and 3 h after intervention, 12 weeks after	Multiplexed NanoString nCounter human miRNA expression assay	26 miRNAs were different between high and low responders, miRNA-136-5p and miRNA-376a-3p were up-regulated both in the acute and chronic treatment
Mueller et al., (2011) [126]	n = 28	80.1 ± 3.7 years, men and women	Old individuals	2 sessions per week for 12 weeks of training with two weekly resistance exercise sessions or eccentric ergometer sessions	Before and 12 weeks after intervention	miRNA analysis by custom-designed low-density PCR arrays	Lower expression of miRNA 1

#### 3.2.4. Epitranscriptomics

In the past, RNA modifications were considered irreversible, and it was believed their function was to give structural stability and catalytic functions. When it was discovered that RNA modifications are reversible, epitranscriptomics began to emerge as a field. Recent studies have provided more information about the location and abundance of RNA modifications. These studies have declared N6-methyladenosine (m6A) as one of the most important RNA modifications.

Literature suggests that m6A is mainly located in the start and stop codons/3′-untranslated regions (3′UTR). Moreover, other RNA modifications have been described such as pseudouridine (Ψ), N1-methyladenosine (m1A), N6,2′-O- dimethyladenosine (m6Am), 5-methylcytosine (m5C) and 5-hydroxymethylcytosine (hm5C), and inosine. Several authors have described other RNA modifications, but they cannot be detected by the current methods. In addition, enzymes or enzymatic complexes which are able to catalyze and remove these RNA modifications (mainly, methyltransferases and demethylases), known as “writers” and “erasers” respectively, remain undisclosed in the majority of cases. One specific role is that of binding proteins, which are able to recognize RNA modification and induct molecular changes; these proteins are called “readers” [127].

**Figure 1:**
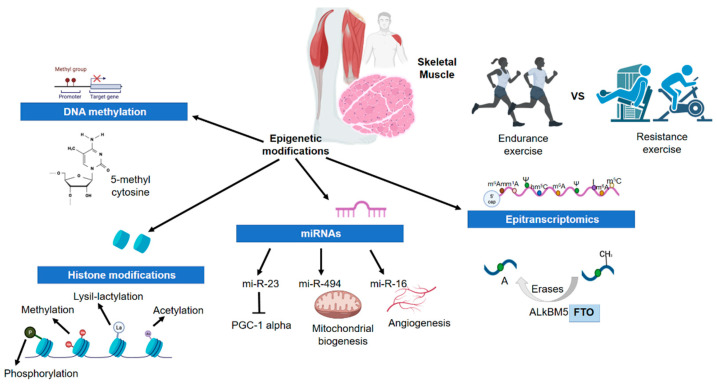
Effects of exercise on epigenetic modifications in skeletal muscle. Abbreviations: FTO, alpha-Ketoglutarate dependent dioxygenase; PGC-1 alpha, Peroxisome Proliferator-Activated Receptor Gamma Coactivator 1 Alpha.

This review has been focused on a known “writer” of m6A in mammalians, methyltransferase 3 (METTL3), which likely plays a relevant role in altering the micro-RNAs’ expression during and after exercise. It is known that METTL3 catalyzes the maturation of micro-RNAs producing an m6A modification in primary micro-RNAs. In skeletal muscle, METTL3 can down-regulate the expression of miR-1, miR-133a, miR-133b, and miR-206; in other words, METTL3 and myomiRs are involved in antagonist functions [128]. For that reason, it is very relevant to know the dynamics of METTL3 and myomiRs deeply. It has been described that after a muscle injury the expression of METTL3 increases, promoting muscle regeneration. Nonetheless, when the muscle cell repair is done, the expression levels of METTL3 decrease, facilitating muscle differentiation [129]. Additionally, METTL3 could play a critical role in skeletal muscle differentiation and regeneration processes according to recent studies. They have demonstrated that after a muscle injury METTL3 and methyltransferase 14 (METTL14), which form a “writer” enzymatic complex, up-regulate the expression of MAPK Interacting Serine/Threonine Kinase 2 (MNK2) through m6A modification to post-transcriptional level. These molecular events could enhance the regeneration and proliferation of the skeletal muscle. The previous modification is mediated by YTH N6-Methyladenosine RNA Binding Protein 1 (YTHDF1), suggesting that it could be a probable “reader”. Consequently, the levels of METTL3/14 and MNK2 decrease, facilitating the differentiation process. The evidence could indicate that MNK2 is an inhibitor of the ERK/MAPK signaling pathway being an essential molecular mechanism in the previously mentioned processes. It is also interesting to mention FTO Alpha-Ketoglutarate Dependent Dioxygenase (FTO) as a known “eraser” of m6A in skeletal muscle (Figure 1). These facts suggest that METTL3/14 could play a key role in controlling the micro-RNAs and other RNA/protein factors activity in the post-training process through m6A modification [129,130]. However, these conclusions should be validated in human cell models because the findings are in mouse cell models, in particular C2C12 cells [129,130].

## 4. Epigenetics Mechanisms in Other Non-Muscle Tissue during Exercise

**Figure 2:**
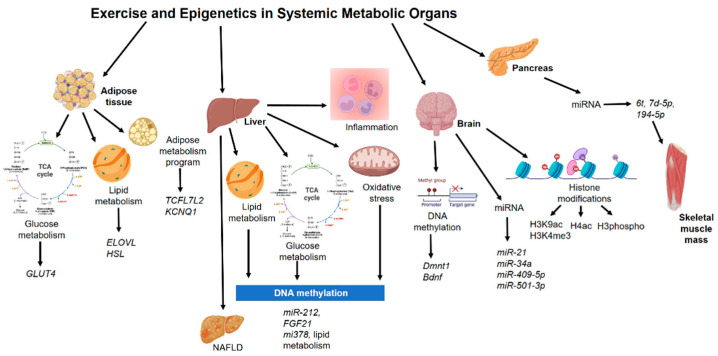
Epigenetics mechanisms in other non-muscle tissue during exercise. Abbreviations: Bdnf, brain-derived neurotrophic factor; Dmnt1, DNA (cytosine-5)-methyltransferase 1; ELOVL, Elongation of very long chain fatty acids protein; FGF21, fibroblast growth factor 21; GLUT4, glucose transporter 4; HSL, hormone-sensitive lipase; KCNQ1, potassium voltage-gated channel subfamily Q member 1; NAFLD, non-alcoholic fatty liver disease; TCFL7L2, Transcription Factor 7-Like 2.

Nowadays, epigenetic changes have become a cause of great interest since it has been shown that external variables, including physical activity and diet, could change the epigenome [131,132]. Transcriptional adaptive responses to exercise happen in several tissues [133]. Previous reports have mentioned that some gene expression alterations after running were observed deeper in the liver than in skeletal muscle in mice [134]. Modifications of histones were reported in several brain regions in response to forced swimming in rats and mice, inducing H3K14 acetylation and serine 10 phosphorylation in spatially distinct regions of the dentate gyrus in a time-dependent manner [135]. Moreover, exercised rats have shown augmented H3 acetylation at the brain-derived neurotrophic factor (BDNF) promoter in the hippocampus [136]. Thus, epigenetic mechanisms are present in other non-muscle tissues during physical activity or exercise (Figure 2).

### 4.1. Adipose Tissue

In chromatin immunoprecipitation assays on human multipotent adipose-derived stem cells, more bindings were identified on *ELOVL* fatty acid elongase 6 functional carbohydrate action [137] than on positive control regions in retinoid-related orphan receptor gamma and thioredoxin-interacting protein, a well-described target of carbohydrate-responsive element-binding protein [138]. Targeting the hormone-sensitive lipase-glucose-responsive transcription factor (ChREBP) interaction may allow therapeutic strategies for the restoration of insulin sensitivity [138].

Adipose altered gene expression in genes associated with carbohydrate metabolism and glucose transport, including glucose transporter (GLUT)-4 has been reported in mice after exercise [139]. Epigenetic analyses have revealed that histone 4 acetylation regulation might be altered on the visceral adipose transcriptome [140]. In particular, histone deacetylase 5 (HDAC5) is implicated as an important mediator of changes in *Glut4* mRNA levels in response to exercise in mice visceral adipose tissue.

DNA methylation on human subcutaneous adipose tissue from 23 healthy men with low physical activity levels, before and after six months of physical activity intervention, and 31 individuals with or without a family history of type 2 diabetes was analyzed. Here, candidate genes had a higher DNA methylation in adipose in response to exercise, including *TCF7L2* and *KCNQ1* [141]. In another study using subcutaneous adipose tissue samples, DNA methylation stimulated by acute exercise was much more predominant in trained versus untrained state with modifications after acute exercise for 32 genes pre-training and 6 post-training, especially at adipocyte-specific genes [142].

### 4.2. Liver

MicroRNAs expression, histone modifications, and DNA methylation by modifying DNA accessibility control the activity of genes related to mitochondrial dysfunction, oxidative stress, lipid metabolism, and inflammation. Studies on liver biopsy samples from patients diagnosed with non-alcoholic fatty liver disease (NAFLD) established the modifications in the methylation of genes associated with mitochondrial biogenesis and lipid metabolism [143].

A pattern of fast food and exercise triggers extensive gene alterations, with enrichment of carbohydrate/lipid metabolic pathways and muscle developmental processes. Hypermethylation at a subset of gene promoters was related to inhibition of tissue development and promotion of carcinogenic processes in mouse liver [144].

In a high-fat diet-induced animal model of NAFLD, exercise was capable of decreasing hepatic overexpression of mir-212, which seemed to be related to lipogenesis and NAFLD development, by targeting FGF-21 [145]. In another study, with a high-fat diet in rats, hypoxic physical exercise decreased hepatic levels of mir-378, related to lipid metabolism regulation and triacylglycerol production [146].

### 4.3. Pancreas

Swimming routine of physical exercise in high-fat diet-fed fathers led to partial inhibition of pancreatic islet morphology modifications and augmented two pancreatic miRNAs (let7d-5p, 194-5p) in male offspring [147]. This study suggests that the profits of the exercise regime reversed the adverse effects on offspring pancreatic dysfunction of high-fat diet consumption in fathers. Here, mice were randomly assigned to a control diet or a high-fat diet for 9 weeks. After the initial feeding period, males fed the high-fat diet were then allocated to one of the following treatments for a further period of 9 weeks: continuation of a high-fat diet or high-fat diet with exercise intervention. Later the founder males had the opportunity to mate with two normal-weight females for a maximum period of 8 days, and at weaning (day 21 of life) male offspring were separated from their mothers, group-housed independently of founder treatment, and maintained on standard chow [147].

### 4.4. Brain

Skeletal muscle initiates crosstalk with other tissues to produce the liberation of protein hormones and myokines, which could exert autocrine, paracrine, and long-distance endocrine effects. In addition, the enhanced release or uptake of metabolites from, and into, contracting muscle cells, respectively, could likewise play the role of a powerful mediator of tissue interactions, in particular, with regards to the central nervous system [148]. Exercised and control rats have shown an increased level and induction of histone H3 phospho-acetylation and c-Fos, and the exercised rats exhibited a greater number of dentate granule neurons expressing the histone changes and initial gene generation [149].

Exercise promotes DNA demethylation in brain-derived neurotrophic factor *(BDNF)* promoter IV and elevates levels of activated methyl-CpG-binding protein 2, as well as BDNF) mRNA and protein in the rat hippocampus. Epigenetic analysis has shown that exercise increases acetylation of histone H3, and protein assessment showed that exercise increases the ratio of acetylated histone–total for histone H3 but had no effects on histone H4 levels [136].

In an animal model experiment using the spontaneous senescence-accelerated P8 mice with exercise, the authors reported modifications in genes related to protein acetylation homeostasis (Sirt1, Hdac6) and Hdac3 and Hdac5 gene expression modulation in the hippocampus. Global histone H3 acetylation scores were diminished in spontaneous senescence-accelerated P8 mouse model mice compared with senescence-accelerated resistant mice [150,151]. In similar studies, long-term exercise in rats and mice enhanced the activity of both histone acetyltransferases (HAT) and histone deacetylases (HDAC) in the hippocampus, which showed improved cognitive function [152,153]. Alterations in the hippocampus following traumatic brain injury showed recuperated hippocampus-related cognitive deficits after running wheel exercise, and both miR-21 and miR-34a were related to the recovery process [154]. Low-capacity runner and high-capacity runner rats were exposed to dietary restrictions. Hippocampus samples have shown that specific binding concerning acetylated histone H3 and BDNF promoters were increased in both groups [155].

Daily exercise protocols induced an increase in histone H4 acetylation levels in prefrontal cortices of 21-month-old rats, without any effects in the young adult group [156]. After 1 week of physical activity, histone 3 acetylation was increased in the hippocampus and cerebellum, and that was correlated with augmented BDNF in the hippocampus. DNMTs and histone deacetylases pattern, important genes that impact DNA methylation and histone changes in general, diminished in hippocampus and cerebellum with exercise [157]. The exercise was also able to increase hippocampal H3K9 acetylation levels in aged rats [158]. A single physical exercise routine reduced DNA methyltransferases levels in the hippocampus of young adult rats [159]. In addition, using a forced exercise protocol the authors have found reduced proinflammatory markers and increased histone H4 acetylation levels in hippocampus 20-month-old rats [160]. Similar results have been found in histone hyperacetylation in the frontal cortex from Wistar rats exposed to a daily running protocol for 2 weeks [161]. DNA methylation in the hippocampus, cortex, and hypothalamus of exercised rats was increased [162].

Exercise was also shown to enrich memory in aged mice, and there was a positive association between 5-methylcytosine miR-137 levels in the hippocampus and hypothalamus and performance in the object location test [163]. In addition, the exercise-induced memory enhancements are complemented by modifications in the hippocampal miRNA-mRNA regulatory net [164]. Different modalities of exercise developed memory performance in aged rats modifying H3K9ac or H3K4me3 at the c-Fos promoter [165]. In 2019, it was reported that DNA methylation in AgRP neurons regulates voluntary exercise behavior in mice [166]. Recently, the beneficial effects in the brain have shown that those modifications might depend on exercise intensity [167] and a short-term period [168]. Here, some microRNAs are described as novel candidates in the influence of exercise on the brain, such as microRNA-409-5p and microRNA-501-3p [169].

In contrast, no effects were observed in DNA methylation in the hippocampus, cortex, hypothalamus, and periaqueductal gray regions of the brain, although it was related to abnormal *Dnmt1* and *Bdnf* expression in cortex, hypothalamus, and periaqueductal gray [170].

## 5. Effects of Metabolism and Its Metabolites on Epigenetic Modifications during Exercise

The process of muscle contraction during exercise requires ATP consumption, and to maintain adequate contractility, an adequate bioavailability of it is needed. Skeletal muscle responds to the high energy demands of exercise, rapidly metabolizing carbohydrates and fats, mainly, and on certain occasions, amino acids. Depending on the manipulation of the training variables, one metabolism will predominate over another, but generally, through resistance training and high-intensity interval training (HIIT), carbohydrate metabolism predominates, and during endurance training at the moderate intensity the fat consumption. Both resistance and endurance training have a great impact on energy production, and consequently, on the generation of metabolites that can epigenetically affect various tissues and organs. As the existence of scientific evidence in this regard in humans is very scarce, most of the studies that will be mentioned are carried out in vitro and experimental animals. When there is a predominance of glycolysis, high amounts of serine are generated, which has been shown to increase methylation in skeletal muscle through recycling of homocysteine to methionine, exerting greater bioavailability to the precursor for de novo methylation. Although it cannot be extrapolated to human skeletal muscle, it has been shown that, in murine model heart muscle, glycolysis regulates a key enzyme for serine resynthesis [171].

As we previously described, lactate is another metabolite with key functions, which increases with the intensity of exercise. Both in vivo and in vitro studies have shown that lactate inhibits HDAC through the histone complex 4, acetylating histone marks, and increasing gene expression in the abovementioned modification called lactylation [112]. Recently, by using stable carbon isotopes, lactyl groups have been identified and it seems to promote epigenetic modifications through lysine residues. Furthermore, lysine lactylation appears to occur in the promoter regions of the encoded genes and is positively correlated with the expression of these transcripts [113,172]. Therefore, lactate is a bioactive metabolite that acts at the systemic level, triggers epigenetic mechanisms of action not only in skeletal muscle, but could also alter the epigenetics of other tissues and organs. Indeed, rats that were treated with lactate were more resilient to stress due to the restoration of HDAC2 and HDAC3 levels in the hippocampus [173]. The study of the effects of lactate produced by exercise on the different epigenetic modifications requires more research, and it is of vital importance to determine specific doses of physical exercise depending on the profile and the individual.

During moderate-intensity resistance training, the main energy substrate predominating is the use of fatty acids. To use them, lipolysis, the metabolic process from which triglycerides are broken down into fatty acids and glycerol, is necessary. Short- or medium-chain fatty acids cross the mitochondrial membrane without the need for any transporter, while long-chain fatty acids require carnitine to enter the mitochondria [174]. Once introduced into the mitochondria, they are used for energy through beta-oxidation. Butyrate, a short-chain fatty acid, has been shown to have an inhibitory effect on HDACs [175], which could prevent and treat diet-induced insulin resistance in mice. In prolonged exercises, acetoacetate is produced by the liver. A minor part of the acetoacetate circulates to the bloodstream, while the majority breaks down into beta-hydroxybutyrate (β-HB), which is released to the blood and can interact in other tissues such as skeletal muscle. β-HB is a ketone body that inhibits HDACs [176], including HDAC 1, 3, and 4, which increases histone acetylation, decreases α-synuclein toxicity, and prevents dopaminergic neurons from cell death through histone acetylation. Therefore, β-HB might regulate HDACs and modify gene expression through these mechanisms. β-HB-mediated inhibition of HDACs in mice also increases BDNF expression, reduces the NAD^+^/NADH ratio, and increases ATP, which is narrowly related to adult neurogenesis [177,178]. Another study revealed that β-HB impacts on gene transcription in rat myotubes, up-regulating PGC1α, CPT1b, mitochondrial sirtuins (SIRT3-5), and the mitochondrial anti-oxidative genes SOD2 and catalase [179] indicating a putative impact on muscle metabolism.

The acetyl-CoA metabolite enters the Krebs cycle and produces different chemical reactions that, during the metabolic process, give rise to intermediates that regulate the activity of different enzymes. After an acute exercise session and a chronic training program, intramuscular concentrations of Krebs cycle intermediates such as fumarate, citrate, or succinate are shown to be higher. In particular, ATP citrate synthase produces epigenetic modifications in skeletal muscle through hyperacetylation of K9, 14, and 27, increasing myoD expression, and pathways associated with IGF-1, an important regulator of muscle cell survival, differentiation, and proliferation [180]. Moreover, ATP citrate synthase plays a role in histone modifications, linking differentiation of satellite cells and myoblasts in skeletal muscle. Therefore, resistance training, which is a great stimulator of IGF-1, could be the main training method that triggers epigenetic adaptations through these mechanisms.

Regarding muscle contraction, calcium is an important molecule that is increased and the intracellular levels produce the binding of the Ca^2+^ ion with calmodulin, forming the CaMK-II complex [181]. Consequently, nuclear activation of CaMK-II phosphorylates MeCP2, a protein that produces epigenetic changes through chromatin remodeling. Thus, the increase in CaMK-IV produces hypomethylation of DNA in skeletal muscle after 7 weeks of resistance training [81], establishing an important epigenetic role for a number of largely unstudied genes in muscle hypertrophy/memory.

Finally, skeletal muscle is an endocrine organ that secretes a large number of cytokines and different types of proteins, in response to muscle contraction, which has an impact not only on the muscle itself but also on a systemic level. Thus, it releases other peptides and nucleic acids called exerkines into the bloodstream. Exerkines can be released from extracellular vesicles known as exosomes, which contain nucleic acids, mRNA, miRNA, and mitochondrial deoxyribonucleic acids [182]. Thus, recent studies have identified that skeletal muscle, through muscle contraction, is also capable of releasing extracellular vesicles into the bloodstream, capable of producing changes in other tissues through miRNAs. These vesicles, together, increase or decrease the expression of certain genes, in addition to other series of post-transcriptional modifications [183]. In particular, resistance exercise causes a higher muscle tissue release of extracellular vesicles containing miR-1, which is captured by white adipose tissue, consequently stimulating lipolysis of this adipose tissue. Therefore, through the endocrine capacity of skeletal muscle through the release of different types of proteins, epigenetic changes can also occur due to the generation of miRNAs that, ultimately, favor metabolic adaptations for the treatment of diseases.

## 6. Conclusions and Future Perspectives

Physical exercise and exercise trigger numerous changes and metabolic adaptations in the organism, resulting in an improvement in functional capacity and health, as well as decreasing the risk of developing metabolic or chronic diseases. In particular, a complex network of molecular mechanisms is activated in skeletal muscle, and the contraction releases active proteins, nucleic acids, and metabolites that may be involved in the inter-organ communication that is likely to mediate many of the effects of exercise. Indeed, some of these metabolites may serve as a substrate to allow epigenetic modifications in skeletal muscle, enabling transcriptional changes to key signaling pathways, and ultimately, muscle adaptation and remodeling. The interaction between these epigenetic modifications and the complexity of physiology is intricate and this should be further researched in order to elucidate all the signal events occurring during exercise in skeletal muscle. Hence, exercise is a powerful tool for altering gene expression profiles in skeletal muscle by epigenetic modifications. DNA hypomethylation and histone hyperacetylation, both in key exercise-responsive genes in skeletal muscle are the most established mechanism of adaptation to exercise. However, other modifications also ensue in histones and epigenetic changes involving microRNAs. Furthermore, the impact of exercise on epigenetic modifications seems to depend on the type, intensity, and duration of exercise. In this context, resistance and endurance exercise differ in the transcriptional regulation in skeletal muscle. In addition, the studies reveal abundant heterogeneity among them in terms of design (e.g., differences between cohorts in sex, age, etc.) and methodology (candidate genes used, type of exercise involved: acute/chronic, resistance/endurance, high/low intensity, training programs of short/long duration); therefore, quantitative analyses of the published literature cannot be performed. Improved understanding of all these interactions would help to optimize the development of novel therapeutic strategies in order to manage metabolic disease through exercise.

## Figures and Tables

**Table 2:** Effects of endurance and resistance training on histone modification in skeletal muscle.

Resistance and Endurance Exercise							
Reference	Sample Size	Age and Sex	Participant Profiles	Exercise Doses	Biopsy Times	Technology	Epigenetic Changes and Gene Expression
McGee et al., (2009) [95]	n = 9	23 ± 1 years, men	Healthy adults	Volume: 60 min, 72 ± 2% VO_2_ max	Before and after exercise	Electrotransference	Higher global acetylation of H3K36
Yu et al., (2003) [96]	n = 9	27 ± 2 years, men	Trained and non-trained individuals	Intensity: 85% VO_2_ max, Rest: 60 s	Before and after exercise	Electrotransference	Higher phosphorylation of H3Histones
